# Quality of Life and Use of Complementary and Alternative Medicines among Narcotics Anonymous Patients: A Cross-Sectional Study in Southeast Iran

**DOI:** 10.1155/2023/3003247

**Published:** 2023-09-11

**Authors:** Mahlagha Dehghan, Hemn Kaka Mirza, Sobhan Alaeifar, Moazemeh Jazinizadeh, Mohammad Hossein Iranmanesh, Fateme Mohammadiakbarabadi, Mina Salehi, Asma Ghonchehpour, Mohammad Ali Zakeri

**Affiliations:** ^1^Department of Critical Care Nursing, Razi Faculty of Nursing and Midwifery, Kerman University of Medical Sciences, Kerman, Iran; ^2^College of Nursing, Al-Kitab University, Kirkuk, Iraq; ^3^Nursing Research Center, Kerman University of Medical Sciences, Kerman, Iran; ^4^Clinical Research Development Unit, Ali-Ibn Abi-Talib Hospital, Rafsanjan University of Medical Sciences, Rafsanjan, Iran; ^5^Social Determinants of Health Research Center, Rafsanjan University of Medical Sciences, Rafsanjan, Iran

## Abstract

**Background:**

Addiction, a chronic and recurrent disorder, is associated with lasting changes in the brain and can significantly affect the quality of life of people. Complementary and alternative medicine (CAM) along with modern medical treatments can improve the quality of life of individuals. This study aimed to investigate the relationship between the use of complementary and alternative medicines (CAMs) and quality of life in narcotics anonymous patients.

**Methods:**

This cross-sectional study was performed on 189 narcotics anonymous patients in southeastern Iran. Using questionnaires such as the demographic information, the World Health Organization Quality of Life-BREF (WHOQOL-BREF), CAM, and satisfaction with the use of CAM, the researcher was able to compile a comprehensive picture of the population.

**Results:**

The mean score of overall quality of life and general health was 64.02 ± 23.32. Overall, 66.1 percent (*n* = 125) of the participants reported using at least one type of CAM in the previous year. Last year, 25.9% of participants used at least one kind of CAM, 22.8% used two types of CAM, 7.9% used three types of CAM, and 4.8% used four to five types of CAM. Thirty-nine point seven percent of them reported using prayer, 36.5% reported using medicinal herbs, 15.3% of participants reported using massage, 14.3% of participants reported using dietary supplements, 12.2% reported using wet cupping, and 8.5% reported using meditation. There were no significant differences in physical, psychological, environmental, and overall quality of life between CAM users and non-CAM users. The prayer users had significantly higher scores in terms of social relationships, environment, and overall quality of life than nonprayer users. Employed participants and opium users had significantly higher overall quality of life than others.

**Conclusion:**

Although there was no difference in quality of life between CAM and non-CAM users, the present study showed that prayer and medicinal herbs were the most commonly used methods among narcotics anonymous patients. However, prayer and religious beliefs were successful in improving the quality of life of these individuals. Physicians and other healthcare providers must advise patients with addiction to use different CAMs in order to improve their quality of life and quit narcotics. Future in-depth studies could help these patients use CAMs and improve their quality of life.

## 1. Introduction

Addiction is a brain disease that is manifested by compulsive substance use despite harmful consequences. Drug addiction is a neuropsychological disorder characterized by a frequent and persistent tendency to use substances despite the harmful consequences [1]. This disease is a fundamental problem for different societies as it affects the lives of millions of people, and huge costs must be spent to fight against it and compensate for its losses [2]. The main clinical signs of addiction include an exaggerated incentive to use substances (craving), impaired self-control (impulsive and compulsive), impaired emotional regulation (negative mood), and increased response to stress [3].

Opioid use disorders (OUDs) are major and long lasting. According to the World Antidoping Agency, 35 million people worldwide suffer from substance use disorders and need medical care [4]. According to official reports in Iran, 2.1% of the population aged 15–64 (1.12 million people) suffer from substance use disorders [5]. Narcotics Anonymous (NA), which was created by persons with addiction to encourage, guide, and provide answers to one another, is one of the best strategies to overcome addiction [6]. Since its inception in 1953, NA has grown to include members from all around the world. Nearly 67,000 NA meetings are held every week in 139 countries throughout the world. This organization helps and supports persons with addiction who wish to quit on their own [7]. Narcotics Anonymous was founded in the Qarchak Rehabilitation Center in Iran in 1990. There is no documentation or exact data about the presence of members or existing patients due to the policy of anonymity. This organization is made up of 29 districts spread throughout 31 provinces [8]. District 9 includes the NA groups of Kerman Province. The first meeting of this district was held in Kerman in 1998, and there are now over 325 groups in district 9, with over 1681 meetings held regularly.

NA meetings were formed to help persons with addiction improve their self-confidence, which may be the most important cause of their temptation [9]. Hosseini et al. studied the quality of life in narcotics anonymous men and found that ongoing participation in the NA meetings significantly improved their quality of life [10].

As social goals become more important, sociology, anthropology, and development programs are used to improve the quality of life of people [11]. Quality of life is a measure to understand how much individuals and groups are satisfied with various aspects of their lives [12]. Quality of life is defined by the World Health Organization (WHO) as individuals' perception of their position in life in the context of the culture and value system in which they live and in relation to their goals, expectations, standards, and concerns [13]. People seeking treatment for substance use disorders ultimately wish to improve their quality of life by reducing or stopping substance use [10]. Studies have shown that poor quality of life in patients with addiction is associated with the physical and psychological consequences of addiction [14]. Researchers and theorists agree that having a poor quality of life is one of the most important reasons why people use drugs again [15].

There are currently various programs for the recovery of patients with addiction. Various factors, including physical exercise along with mental programs, can affect the quality of life and improve mental disorders [16]. Although pharmacotherapy can reduce opioid overdose, methadone and buprenorphine are both legally restricted, particularly in rural areas. Individuals may be forced to adopt complementary and alternative treatments for opioid use disorders (OUDs) if the supply of opioids prescribed for nonmedical use declines [17].

Complementary and alternative medicine (CAM) is used in addition to modern medical treatments for specific or general health conditions [18]. CAM has over 1800 types [19]. Over 100 million Europeans use CAM [20]. Any activity that replaces medical therapy is known as an alternative, whereas any action that supplements medical care is known as complementary [21]. Over the past three decades, there has been a great deal of interest in using acupuncture to treat substance abuse all over the world [22]. Many patients use vitamins, minerals, and herbs as part of their care without consulting with their doctors. There is evidence that CAM products can be safe, effective, and cost-effective when used properly; however, adverse effects [23, 24] and drug interactions can occur when CAM products are misused [25].

According to the review of the literature, no study addressed the relationship between the use of complementary medicine, relapse to substance use, and quality of life among patients with addiction. Stomski et al. conducted a prospective cohort study in Australia and found that a complementary intervention significantly increased the quality of life of cancer patients and reduced their restlessness [26]. Dehghan et al. found that users of CAM methods, including relaxation and meditation techniques, had a higher quality of life than non-CAM users [19].

The NA includes a wide range of people and has a profound effect on other walks of life. However, limited studies have been conducted on the health and quality of life of these people. Patients' relapse to substance use is often caused by temptation, which is affected by many factors. Therefore, further studies should be done on the quality of life and the temptation to use substances among individuals with addiction. Therefore, this study aimed to investigate the relationship between the use of CAM and quality of life in patients with addiction.

## 2. Methods

### 2.1. Study Design and Setting

This is a cross-sectional study on the relationship between quality of life and use of CAM in the narcotics anonymous population in Iran. The data were collected from August to November 2021.

### 2.2. Sampling and Sample Size

After obtaining the necessary permits, the researcher first coordinated with anonymous persons with addiction in Kerman. Then, he identified the eligible individuals according to the entry criteria and invited them to participate in the study. The researcher refers to the association of anonymous persons with addiction and starts sampling by available methods. The study population in this study was all men and women attending NA meetings. Inclusion criteria included diagnosis of substance dependence, an age over 18 years, and literacy. Exclusion criteria included incomplete completion of the questionnaire. We used Cochran's formula for an infinite population to estimate the sample size (*Z* = 1.96, *d* = 0.07, and *n* = 196). According to dropout probability, 250 questionnaires were distributed. The researcher approached 321 participants to complete the questionnaire. Of the 311 participants, 17 participants were excluded from the study due to their age of less than 18 years, 23 participants due to illiteracy, and 31 participants due to unwillingness to cooperate. After collecting the questionnaires, 250 questionnaires were collected, of which 61 questionnaires were excluded from the study due to noncompletion. The response rate was 75.6%. Finally, 189 participants completed the questionnaire.

### 2.3. Ethical Issue

The ethics committee of Kerman University of Medical Sciences approved the study protocol (IR.KMU.REC.1399.657). Participants signed an informed consent form before beginning the research. The study's objectives, confidentiality, and anonymity were described, and volunteers were given full authority to complete the questionnaire.

### 2.4. Questionnaires

#### 2.4.1. Demographic Information Questionnaire

The demographic and clinical information questionnaire included age, sex, marital status, education level, job, living place, age of first substance use, history of substance use, history of withdrawal from a substance, being an NA member, history of mental disorders, history of physical disorders, history of opium usage, history of different substances used, history of a family member's addiction, and history of relapse to substance use.

#### 2.4.2. WHOQOL-BREF Questionnaire

This questionnaire, developed by the World Health Organization, comprises 26 questions. WHOQOL-BREF covers four domains of physical health, psychological health, social relationships, and environment in a five-point Likert scale [27]. There are two separate questions, which ask specifically about (1) the individual's overall perception of their health and (2) the individual's perception of their quality of life. Questions 3, 4, and 26 are scored in reverse. Following the completion of the relevant computations in each domain, a score ranging from 4 to 20 will be obtained in each domain independently, which can be converted to 0–100. Nejat et al. evaluated the validity and reliability of this questionnaire. Using the intraclass correlation index, the reliability scores for physical health, psychological health, social relationships, and environment were 0.77, 0.77, 0.55, and 0.84, respectively [28].

#### 2.4.3. Complementary and Alternative Medicine Questionnaire

Dehghan et al. developed this questionnaire, which is about the application of CAM and includes 10 questions on the use of some types of CAM (herbal medicines, wet cupping, dry cupping, massage, dietary supplements, acupuncture, acupressure, homeopathy, relaxation techniques such as yoga, and prayers (five times every day at prescribed times)) [29]. Each one is scored based on a yes/no scale. Participants were asked how often they performed prayers (several times a year = 1 to every day = 6). Reasons for using CAM were also measured using four options: treatment of addiction, reduction of physical complications of the withdrawal, reduction of mental complications of the withdrawal, and others. To determine the validity, the questionnaire was given to 10 faculty members of the Razi School of Nursing and Midwifery in Kerman, and the content validity index of the questionnaire was calculated to be 0.96. Cronbach's alpha coefficient was 0.85 [19].

#### 2.4.4. Satisfaction with the Use of Complementary Medicine Questionnaire

Ghaedi et al. developed this questionnaire in Iran. This questionnaire consists of 9 items regarding access to the method, ease of use, harmlessness, noninterference with daily activities, reduction of physical and mental symptoms, noninterference with other treatments, recommendation of the method to others, and affordability (from completely satisfied = 4 to completely dissatisfied = 0) [30]. The score of satisfaction with complementary medicine was between 0 and 36, with a higher score indicating more satisfaction (minimum 9 and maximum 36). The validity of the questionnaire in Ghaedi et al.'s studies was obtained using face and content validity, and its internal consistency was obtained to be 0.85 using Cronbach's alpha coefficient [30].

### 2.5. Data Collection

The researcher identifies the eligible individuals and invites them to participate in the study. In the individual and separate interview, the objectives of the research, the method of its implementation, and the method of filling out the questionnaire are explained to each sample, and if desired, they are asked to participate in the study. The study's objectives and methodology were presented to the participants, and their written consent was obtained.

### 2.6. Data Analysis

Descriptive and inferential statistics as well as SPSS 25 were used to analyze the data. Descriptive statistics were used to describe demographic characteristics and mean scores. To determine the relationship between the quality of life and CAM questionnaires, the independent *t*-test was used. To determine the relationship between the overall quality of life and demographic and clinical characteristics, the Mann–Whitney *U* test, independent *t*-test, and analysis of variance were used. A multiple linear regression model was used to determine the predictors of overall quality of life. A significance level less than 0.05 was considered.

## 3. Results

### 3.1. Participants' Demographic and Clinical Characteristics

The mean age of the participants was 38.24 ± 11.18 years (min = 19 and max = 75). The majority of the samples were male, married, educated, and employed (Table 1). The data related to substance use and withdrawal are presented in Table 2.

### 3.2. Quality of Life in the Narcotics Anonymous Population

The mean scores of physical health, psychological health, social relationships, environment, and overall quality of life and general health were 60.20 ± 18.12, 55.84 ± 13.98, 61.11 ± 21.57, 53.64 ± 14.69, and 64.02 ± 23.32, respectively. Therefore, the highest score belonged to the physical health while the lowest belonged to the environment. The mean scores of all domains of quality of life were above the scale midpoint of 50.

### 3.3. CAM Uses and Satisfaction in the Narcotics Anonymous Population

Overall, 66.1 percent (confidence interval: 59.3–72.5% and *n* = 125) of the participants reported using at least one type of CAM in the previous year. In addition, 25.9 percent (*n* = 49) of the individuals used only one form of CAM, 22.8 percent (*n* = 43) used two types of CAMs, 7.9 percent (*n* = 15) used 3 types of CAMs, 4.8 percent (*n* = 9) used 4 types of CAMs, and 4.8 percent (*n* = 9) used five to nine types of CAMs in the last year. Thirty-nine point seven percent (*n* = 75) reported using prayers, 36.5% (*n* = 69) reported using medicinal herbs, 15.3% of the participants (*n* = 29) reported using massage, 14.3% of the participants (*n* = 27) reported using dietary supplements, 12.2% (*n* = 23) reported using wet cupping, 8.5% (*n* = 16) reported using meditation, 6.3% (*n* = 12) reported using homeopathy, 5.3% (*n* = 10) reported using dry cupping, 4.8% (*n* = 9) reported using acupuncture, and 2.6% (*n* = 5) reported using acupressure (Table 3). The common reasons for using CAM are presented in Table 3. The mean score of satisfaction with CAM use was 22.0 ± 6.72 (min = 2 and max = 36), which was above the scale midpoint of 18.

### 3.4. The Association between Quality of Life, CAMs Use, and Other Study Variables in Narcotics Anonymous Patients

There were no significant differences in physical health, psychological health, environment, and overall quality of life between the CAM users and non-CAM users; however, the score of social relationships was significantly higher in the CAM users compared with the non-CAM users (Table 4). When analyzing each type of CAMs separately, the prayer users had significantly higher scores in social relationships, environment, and overall quality of life than nonprayer users. In addition, the medicinal herb users had a significantly higher score for social relationships than nonmedicinal herbs users (Table 5). There was no association between other types of CAMs and different aspects of quality of life (*P* > 0.05). Among other study variables, only employed participants and participants with a history of opium and its derivatives usage had significantly higher overall quality of life than others. In addition, participants with history of marijuana usage had lower overall quality of life than others (Tables 1 and 2). For further analysis, multiple linear regression was conducted, and the overall quality of life score was considered as dependent variable while history of opium and its derivatives usage (yes/no), history of marijuana usage (yes/no), job, medicinal herbs users (yes/no), and prayer users (yes/no) were considered as independent variables. The results showed that history of opium usage, job, and prayer users were predictors of overall quality of life among patients (Table 6).

## 4. Discussion

To our knowledge, this is the first study that determines the relationship between the use of CAM, quality of life, and the temptation to use substances in narcotics anonymous individuals. The present study found that 66.1% of the participants used at least one type of CAM in the past year. In addition, 25.9% of the participants used only one type of CAM and 22.8% used two types of CAM. Furthermore, Sylvain et al. studied patients with alcohol or tobacco disorders in Switzerland and showed that 62.5% used CAM for comorbidities and not specifically for substance use disorders (SUDs). Almost everyone wanted CAM to be part of the healthcare system, and thought that if they needed to see a CAM specialist, their general practitioner should refer them [31].

However, previous studies reported less use of CAM. Woodward et al. found that 34% of the adults with mood, anxiety, or substance use disorders used at least one type of CAM in the past 12 months [32]. Manheimer et al. reported that 45% of the intravenous drug users applied at least one type of CAM [33]. These results suggest that as people gain access to the Internet and mass media, they may learn more about CAM usage. Jazieh et al. studied the use of CAM in cancer patients from 2006 to 2018 and showed that 78.9% of them used CAM from 2006 to 2008 and 96.8% used CAM from 2016 to 2018 [34].

A review of the literature suggests the use of CAM to reduce the anxiety caused by diseases [29], the signs and symptoms of diseases [35], and other problems, such as back pain, depression, insomnia, severe headaches or migraines, and stomach or intestinal illnesses [36]. However, people struggling with addiction may use CAM to reduce the problems caused by addiction and its complications. Sivertsen et al. showed that women who drank alcohol frequently were more likely to use natural medicines and self-medication techniques (meditation, yoga, chi gong, or tai chi) and visit a CAM doctor. Both men and women were more likely to use CAM methods to reduce alcohol-related injuries [37]. Manheimer et al. found that having a higher education and lower self-rated health were the two strongest predictors of CAM use in intravenous drug users. There was a high level of self-perceived effectiveness of CAM therapies, and CAM users were likely to use CAM for reasons related to their addiction [33]. Injuries from addiction and substance use can increase the need for medical services and pain relief, thus increasing the use of CAM techniques. However, it is necessary to pay attention to the differences in societies' consumption patterns and cultures regarding substance use.

According to some research studies, improving understanding of CAM might potentially simplify the admission of people with drug use disorders into healthcare and lessen the therapeutic gap in patients with SUD. Research on CAM usage among patients with SUD should not only focus on reducing substance use but also on improving associated disorders and symptoms [31]. Therefore, the healthcare system must need to try to learn more about how CAM is used by people with addiction. This will help reduce injuries and improve related disorders and symptoms.

In the present study, prayer (39.7%) and medicinal plants (36.5%) were the most commonly used CAM methods in the past year. Manheimer et al. also studied intravenous drug users and showed that the top three therapies—religious healing, relaxation techniques, and meditation—were all from the mind-body domain [33]. Substance use disorders are chronically recurrent and lead to significant impairments in psychosocial function. Conventional treatments had no effect on the outcome of these disorders, and relapses continued despite the development of newer drugs [38]. As a result, there is a focus on CAMs. However, Behere et al. showed that only acupuncture, herbal therapies, and mind-body interventions had positive results in human experiments and were promising for the future [39]. The results of studies conducted on other groups were different from the results of the present study. Dehghan et al. showed that dietary supplements (61.3%), prayer (57.9%), and herbal medicines (48.8%) were the most common CAM methods used by the general population in the COVID-19 epidemic [29]. In addition, the most common types of CAMs used by hemodialysis patients were prayer and herbal medicines [19]. Most of the terminally ill cancer patients used prayer (83.7%) and herbal medicines (35.8%) [40, 41], which is consistent with the use of herbal medicines in the present study.

The general belief in the use of CAM should be considered in interpreting the results. Sylvain et al. studied patients with alcohol or tobacco disorders in Switzerland and showed that hypnosis was higher in patients seeking help to quit smoking than in those with alcohol because the public believed that hypnosis was effective in quitting smoking [31]. Different societies may have different beliefs in the use of CAM. It is clear that various factors play a role in the use of CAM. Due to the specific conditions of patients with addiction, the different effects of opioids, and many challenging factors in the lives of these people, future studies must focus on the conditions of persons with addiction in using CAM.

The present study showed no significant difference in overall quality of life between CAM and non-CAM users. In addition, Dehghan et al. showed no difference in physical and psychological symptoms between CAM and non-CAM users with cancer [40]. However, Dehghan et al. showed that hemodialysis patients, who used some types of CAM, including relaxation and meditation techniques, had a higher quality of life than those who did not use CAM [19]. Manheimer et al. found that having a higher education and lower self-rated health were the two strongest predictors of CAM use among intravenous drug users. There was a high level of self-perceived effectiveness of CAM therapies, and CAM users were likely to use CAM for reasons related to their addiction [33]. However, substance use disorders are increasingly considered as chronic conditions. Substance use disorders (SUDs) are defined as maladaptive patterns of substance use that lead to clinically significant distress and potentially affect physical or mental functioning. Substance abusers seek help to escape these negative consequences, achieve a better life, and improve their quality of life [42]. Therefore, people with addiction may use CAMs to increase their quality of life. However, evidence suggests that CAM users tend to have more than one medical condition and may have more certain conditions or consider their general health poorer than non-CAM users. Studies show that both demographic and health characteristics are independently involved in the use of CAM. As a result, demographic characteristics and health-related factors are related to using CAM [43]. Future research is needed to address the methodological limitations of existing studies. We did not find any further studies in this regard, so results should be interpreted with caution.

Another important finding of the present study was the high overall quality of life of employed participants and opium users. In addition, marijuana users had a lower overall quality of life than others did. However, Marini et al. (2013) showed that poor quality of life was associated with unemployment, more years of study, use of medication, and individual visits with a specialist among alcohol and other drug users. The severity of depressive symptoms is also an independent determinant of quality of life disorder, which predicts more than 50% of physical and psychological changes [44]. In addition, Muller et al. (2016) showed a strong relationship between depression and poor quality of life in women with SUD, as well as between physical inactivity, very low weight, and very poor quality of life in men. Methadone/buprenorphine use was also a protective factor for men who reported poor and very poor quality of life [45]. Examining the factors related to quality of life among people with addiction is very important to determine effective interventions. In addition to focusing on quality of life- and drug-related factors, treatment should target persons with addiction with these specific vulnerabilities, and proven interventions should be considered to improve their quality of life.

According to the present study, participants who performed prayers had higher scores in terms of social relationships, environment, and overall quality of life than those who did not perform prayers. Qureshi et al. also showed that due to the positive effects of religious and spiritual therapies on health, well-being, disease control, and quality of life around the world, CAM physicians and the public were very interested in spiritual therapies [46]. However, Woodward et al. found that among adults with mood, anxiety, or substance use disorders, a small number of whites reported prayer and other spiritual practices (47%) compared to African Americans (63%) and African Caribbean people (68%) [32]. The results show that cultures and communities can affect the amount and type of CAM use. As Iran is an Islamic country, it was predictable that a high percentage of people would use spirituality and prayer to increase their quality of life.

These findings generally suggest that although there are differences in the use of CAM among individuals, there are important differences among persons with addiction that may be rooted in their specific and critical circumstances and different consumption patterns. Since CAMs come from different cultures, it is important to understand how different cultural groups use CAMs in common healthcare systems. Although this study focused specifically on patients with addiction, further study on the use of CAM among diverse groups of persons with addiction is needed to improve their quality of life.

Our study had several limitations. First, since this cross-sectional study did not examine the cause-and-effect relationship, it is suggested that future research be in the form of an interventional or longitudinal study. Second, since the participants of the present study were addicted patients in southeastern Iran, caution should be exercised in generalizing the results. In addition, as the present study was done in the Islamic Republic of Iran, it must be considered in future studies. Paying attention to the specific situation of addicted people and various types of addiction can affect the variables studied in these patients, so it is necessary to focus on the status of patients and the type of addiction. Furthermore, as the population was N/A and all of them were in the withdrawal time (at least one month has lasted from their withdrawal), we did not ask about the current substance use. Therefore, this issue should be considered in future studies. Finally, the existence of few research studies that particularly explored the association between CAM usage and quality of life in addicted patients suggest that this topic requires additional study.

## 5. Conclusion

This study showed that the use of CAM was common among narcotics anonymous patients and prayer and medicinal herbs were the most commonly used methods. However, there was no difference in quality of life between CAM and non-CAM users. Prayer users had higher scores than nonprayer users in terms of social relationships, environment, and quality of life, indicating the impact of religious beliefs on increasing the quality of life of persons with addiction. In addition, employed participants and opium users had a higher quality of life. In examining the quality of life of addicted people, it is necessary to pay attention to various factors that are rooted in their specific and critical conditions and different consumption patterns. Since different cultures influence CAM use differently, understanding these differences can help patients with addiction use CAMs and improve their quality of life. When advising patients to improve their living conditions and stop taking drugs, physicians and other healthcare practitioners should be taught about their patients' interest in various CAMs. Future studies could pave the way for CAMs to improve the quality of life of patients with addiction.

## Figures and Tables

**Table 1:** Demographic characteristics of the participants and their relation with overall quality of life among the participants.

Variable	Frequency (percent)	Overall quality of life		Statistical test (*P* value)
		Mean	SD	
Age (years)				
≤30	53 (28.0)	59.67	26.93	*F* = 2.0 (0.12)
31–40	69 (36.5)	64.49	21.18	
41–50	43 (22.8)	70.64	17.86	
>50	24 (12.7)	60.42	27.50	
Sex				
Female	27 (14.3)	58.33	24.76	*t* = −1.37 (0.17)
Male	162 (85.7)	64.97	23.01	
Marital status				
Single	52 (27.5)	60.58	22.60	*F* = 0.78 (0.46)
Married	124 (65.6)	65.32	22.64	
Divorced/widow(er)	13 (6.9)	65.38	31.93	
Education level				
<Diploma	84 (44.5)	65.77	22.05	*F* = 0.60 (0.55)
Diploma	60 (31.7)	61.46	24.06	
Academic	45 (23.8)	64.17	24.80	
Job				
Employed	158 (16.4)	66.53	21.69	*t* = −3.44 (0.001)
Unemployed	31 (83.6)	51.21	27.26	
Living place				
Urban	165 (87.3)	64.09	24.43	*t* = 0.11 (0.91)
Rural	24 (12.7)	63.54	13.75	

SD: standard deviation, *t*: independent *t*-test, and *F*: analysis of variance.

**Table 2:** Clinical characteristics of the participants and their relation with overall quality of life using among the participants.

Variable	Frequency (percent)	Overall quality of life		Statistical test (*P* value)
		Mean	SD	
Age of first drug use (years)				
≤18	68 (36.0)	65.44	22.97	*F* = 1.36 (0.26)
19–30	111 (58.7)	64.19	23.38	
>30	10 (5.3)	52.50	24.15	
History of drug use (years)				
<5	45 (23.8)	61.67	25.48	*F* = 0.72 (0.49)
5–10	62 (32.8)	62.70	22.0	
>10	82 (43.4)	66.31	23.13	
History of withdrawal (years)				
<5	124 (65.6)	62.90	23.48	*F* = 1.37 (0.26)
5–10	46 (24.3)	63.59	19.90	
>10	19 (10.1)	72.37	29.34	
Being an NA member (years)				
<5	113 (59.8)	62.50	23.02	*F* = 2.97 (0.05)
5–10	51 (27.0)	62.25	22.98	
>10	25 (13.2)	74.50	23.52	
History of mental disorders				
Yes	24 (12.7)	59.38	21.25	*t* = −1.04 (0.30)
No	165 (87.3)	64.70	23.59	
History of physical disorders				
Yes	28 (14.8)	66.96	25.51	*t* = 0.72 (0.47)
No	161 (85.2)	63.51	22.96	
History of opium^*∗*^ and its derivatives usage				
Yes	138 (73.0)	68.21	20.71	*t* = 4.24 (<0.001)
No	51 (27.0)	52.70	26.26	
History of glass/ice				
Yes	71 (37.6)	60.03	26.53	*t* = −1.83 (0.07)
No	118 (62.4)	66.42	20.91	
History of crack				
Yes	17 (9.0)	58.82	29.24	*t* = −0.96 (0.34)
No	172 (91.0)	64.53	22.69	
History of marijuana use				
Yes	32 (16.9)	55.86	23.76	*t* = −2.19 (0.03)
No	157 (83.1)	65.68	22.95	
History of cocaine use				
Yes	11 (5.8)	57.95	37.61	*Z* = −0.43 (0.67)
No	178 (94.2)	64.40	22.25	
History of other drug uses				
Yes	53 (28.0)	62.03	28.79	*Z* = −0.04 (0.96)
No	136 (72.0)	64.80	20.87	
History of family member/close friend/close relatives addiction				
Yes	152 (80.4)	64.56	23.66	*t* = 0.64 (0.52)
No	37 (19.6)	61.82	22.04	
History of unsuccessful withdrawal				
Yes	158 (83.6)	65.19	24.04	*t* = 1.56 (0.12)
No	31 (16.4)	58.06	18.41	

SD: standard deviation, NA = narcotics anonymous, *t* = independent *t*-test, *F* = ANOVA, and *Z* = Mann–Whitney U. ^*∗*^Opium is dried latex obtained from the seed capsules of the opium poppy *Papaver somniferum*.

**Table 3:** The use of CAMs and the reasons for using each type of CAMs among the participants.

Variable	Frequency of the users (%)	95% confidence interval of percentage (%)	Reasons for using the CAM methods (*n* (%)^*∗*^)			
			Addiction treatment	Reducing physical complications of the withdrawal	Reducing mental complications of the withdrawal	Others
Medicinal herbs	69 (36.5)	29.6–43.4	3 (1.6)	31 (16.4)	7 (3.7)	28 (14.9)
Dry cupping	10 (5.3)	2.1–9.0	—	6 (3.2)	—	4 (2.1)
Wet cupping	23 (12.2)	7.4–16.9	2 (1.1)	7 (3.7)	—	14 (7.4)
Massage	29 (15.3)	10.6–21.2	—	3 (1.6)	—	14 (7.4)
Dietary supplements	27 (14.3)	9.5–19.6	—	7 (3.7)	6 (3.2)	14 (7.4)
Acupunture	9 (4.8)	1.6–7.9	2 (1.1)	2 (1.1)	1 (0.5)	4 (2.1)
Acupressure	5 (2.6)	0.5–5.3	2 (1.1)	1 (0.5)	—	2 (1.0)
Homeopathy	12 (6.3)	3.2–10.1	1 (0.5)	1 (0.5)	4 (2.1)	6 (3.2)
Meditation	16 (8.5)	4.8–12.7	—	2 (1.1)	7 (3.7)	7 (3.7)
Prayer	75 (39.7)	32.8–46.6	1 (0.5)	2 (1.1)	32 (16.9)	41 (21.7)

^*∗*^Missing value; CAM: complementary and alternative medicines; others: strengthening the immune system, decreasing fatigue.

**Table 4:** The quality of life and its association with CAM usage among the participants.

Variable	CAM user		Independent *t*-test (*P* value)
	Yes (mean/SD)	No (mean/SD)	
Physical health domain	61.09 ± 16.24	58.48 ± 21.34	−0.94 (0.35)
Psychological health domain	57.03 ± 13.20	53.52 ± 15.22	−1.64 (0.10)
Social relationships domain	63.33 ± 20.47	56.77 ± 23.14	−2.0 (0.048)
Environment domain	54.68 ± 14.34	51.61 ± 15.25	−1.36 (0.18)
Overall quality of life	66.50 ± 20.55	59.18 ± 27.48	−1.88 (0.06)

SD: standard deviation.

**Table 5:** The quality of life and its association with prayer and medicinal herbs usage among the participants.

Variable	Prayer user		Independent *t*-test (*P* value)
	Yes (mean/SD)	No (mean/SD)	
Physical health domain	62.05 ± 17.18	58.99 ± 18.68	−1.14 (0.26)
Psychological health domain	56.83 ± 13.24	55.19 ± 14.46	−0.79 (0.43)
Social relationships domain	65.67 ± 17.22	58.11 ± 23.61	−2.38 (0.02)
Environment domain	56.88 ± 13.22	51.51 ± 15.26	−2.49 (0.01)
Overall quality of life	68.83 ± 20.17	60.86 ± 24.75	−2.33 (0.02)

	Medicinal herbs user		Independent *t*-test (*P* value)
	Yes (mean/SD)	No (mean/SD)	

Physical health domain	61.54 ± 16.61	59.43 ± 18.95	−0.77 (0.44)
Psychological health domain	56.34 ± 14.18	55.56 ± 13.91	−0.37 (0.71)
Social relationships domain	66.18 ± 19.38	58.19 ± 22.29	−2.48 (0.01)
Environment health domain	56.67 ± 13.14	52.11 ± 15.35	−1.90 (0.06)
Overall quality of life	68.11 ± 18.51	61.67 ± 25.45	−1.84 (0.07)

SD: standard deviation.

**Table 6:** A multiple regression analysis summary for overall quality of life.

Variable	Unstandardized coefficients		Standardized coefficients	*t*	*P* value	95% confidence for *B*	
	*B*	Standard error	Beta			Lower bound	Upper bound
Constant	44.02	4.35		10.12	<0.001	35.44	52.60
History of opium and its derivatives usage	11.76	3.84	0.22	3.06	0.003	4.18	19.34
Job	10.42	4.58	0.17	2.27	0.02	1.38	19.46
Prayer users	6.81	3.28	0.14	2.08	0.04	0.34	13.72

Adjusted *R*^2^ = 0.12, *F* = 9.29, and *P* < 0.001.

## Data Availability

The data used to support the findings of this study are made available from the corresponding author upon reasonable request.

## Abbreviations

CAM: Complementary and alternative medicine
CAMs: Complementary and alternative medicines
NA: Narcotics Anonymous
OUDs: Opioid use disorders
SUDs: Substance use disorders
WHO: World Health Organization
WHOQOL-BREF: World Health Organization Quality of Life-BREF.
